# A dataset of new occurrence records of primates from the arc of deforestation, Brazil

**DOI:** 10.5194/pb-11-1-2024

**Published:** 2024-01-22

**Authors:** Rodrigo Costa-Araújo, Gustavo Rodrigues Canale, Fabiano Rodrigues de Melo, Raimundo Rodrigues da Silva, Ivan Batista da Silva, Raony Macedo de Alencar, Luciano Ferreira da Silva, Leandro Jerusalinsky, Renata Bocorny de Azevedo, Eduardo Marques Santos Júnior, Italo Mourthé, Emil José Hernández Ruz, José de Sousa e Silva-Jr., Christian Roos, Izeni Pires Farias, Tomas Hrbek

**Affiliations:** 1 Primates Genetics Laboratory, German Primate Center, Leibniz Institute for Primate Research, 37077 Göttingen, Germany; 2 Sinop Applied Ecology Group, Centre of Biodiversity Studies of the Southern Amazon, Federal University of Mato Grosso, Sinop, 78556-706, Brazil; 3 Department of Forestry Engineering, Federal University of Viçosa, Viçosa, 36570-900, Brazil; 4 Mastozoology collection, Goeldi Museum, Belém, 66077-830, Brazil; 5 Amazon Marmosets Project, German Primate Center, Leibniz Institute for Primate Research, 37077 Göttingen, Germany; 6 Alcoa World Alumina Brasil, Juruti, 68170-000, Brazil; 7 Centro Nacional de Pesquisa e Conservação de Primatas Brasileiros, Instituto Chico Mendes de Conservação da Biodiversidade, Cabedelo, 58108-012, Brazil; 8 Gerência Regional Nordeste, Instituto Chico Mendes de Conservação da Biodiversidade, Cabedelo, 58108-012, Brazil; 9 Biology and Conservation of Primates Research Group, Mamirauá Institute for Sustainable Development, Tefé, 69553-225, Brazil; 10 Zoology Laboratory, Federal University of Pará, Altamira, 68372040, Brazil; 11 Gene Bank of Primates, German Primate Center, Leibniz Institute for Primate Research, 37077 Göttingen, Germany; 12 Evolution and Animal Genetics Laboratory, Federal University of Amazonas, Manaus, 69077-000, Brazil; 13 Department of Biology, Trinity University, San Antonio, Texas 78212-7200, United States

## Abstract

​​​​​​​The so-called arc of deforestation is a major agricultural and industrial frontier in southern Amazonia and northern Cerrado of Brazil. As arboreal mammals, the primates in this region are therefore threatened by forest loss and fragmentation. At the same time, knowledge about the taxonomic diversity and distribution ranges of these taxa is incomplete, which might hamper efficient conservation measurements. New species have been recently discovered in this region, and their ranges remain imprecise because only a few occurrence records are available for each species. Here we present 192 new records of 22 species and subspecies of *Alouatta*, *Aotus*, *Ateles*, *Cebus*, *Chiropotes*, *Lagothrix*, *Leontocebus*, *Pithecia*, *Plecturocebus*, *Saimiri*, and *Sapajus*, collected in 56 different localities during 10 field expeditions across the arc of deforestation between 2015 and 2018. Based on these new records, we extend the ranges of *Alouatta puruensis*, *Ateles chamek*, and *Saimiri collinsi*; identify potential hybridization zones between *A. puruensis* and *A. discolor*, and between *At. chamek* and *At. marginatus*; redefine the range of *Plecturocebus moloch*; and clarify the ranges of *P. baptista* and *P. hoffmannsi*. Moreover, these results and the dataset are valuable for further research on, for example, species distribution and habitat use modeling, for assessing species extinction risks, and for supporting efforts for the conservation of species increasingly threatened on a global deforestation frontier.

## 1 Introduction

The arc of deforestation is a major agricultural and industrial frontier moving north through southern Amazonia and the northern Cerrado (tropical savanna) of Brazil across the southern portions of the states of Pará and Amazonas, in Rondônia, and in the north of the state of Mato Grosso (Fearnside et al., 2009; Silva Junior et al., 2019). The arc of deforestation accounts for approximately half of the global forest conversion into anthropogenic landscapes between 1990 and 2015 (FAO, 2016). This is a direct consequence of the implementation of the BR-230 (Transamazônica) and BR-163 (Cuiabá-Santarém) roads in the early 1970s, which prompted an exponential and disorderly invasion of people from southern and southeastern Brazil motivated by land tenure opportunities and the lack of enforcement of environmental laws for the production of beef, soy bean, and corn (Laurance et al., 2002; Kirby et al., 2006; Fearnside, 2017).

The arc of deforestation still harbors a rich but little-known diversity of primates. Fifty-two primate species are known to occur in this region, including three described recently: *Mico munduruku* Costa-Araújo, Farias, and Hrbek, 2019 (Costa-Araújo et al., 2019); *Mico schneideri* Costa-Araújo, Silva-Jr., Boubli, Rossi, Hrbek, and Farias, 2021 (Costa-Araújo et al., 2021); and *Plecturocebus grovesi* Boubli, Byrne, M. N. F. Silva, Silva-Jr., Costa-Araújo, Bertuol, Gonçalves, Melo, Rylands, Mittermeier, F. E. Silva, Nash, Canale, Alencar, Rossi, Carneiro, Sampaio, Farias, Schneider, and Hrbek, 2019 (Boubli et al., 2019). These primates are directly and negatively affected by the degradation, fragmentation, and loss of habitat. The conservation status of *M. munduruku*, *M. schneideri*, *P. grovesi* (Boubli et al., 2020; Costa-Araújo et al., 2020, 2022a), and others, such as *Ateles marginatus*, *Cebus kaapori*, *Chiropotes satanas*, *Mico marcai*, and *Plecturocebus vieirai*, is rapidly escalating to threatened as assessed on the IUCN Red List of Threatened Species (Silva et al., 2020; Fialho et al., 2021; Port-Carvalho et al., 2021; Ravetta et al., 2021; Costa-Araújo et al., 2022b, c). In 2022, *P. grovesi* was listed as one of the world's 25 Most Endangered Primates (Boubli et al., 2022).

**Figure 1:**
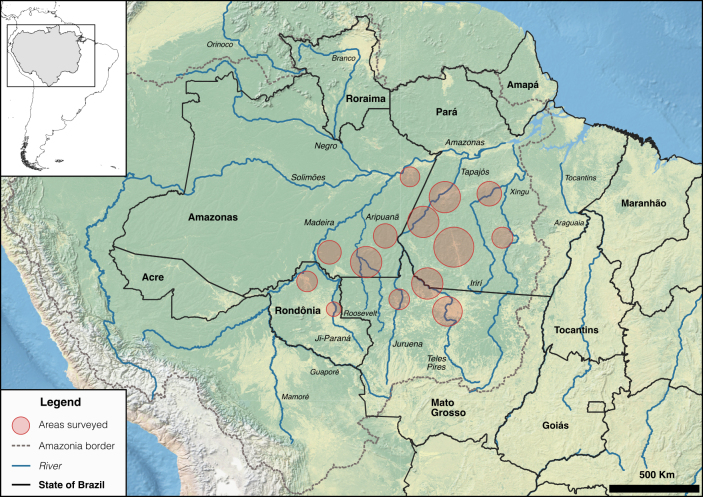
Areas surveyed for primate occurrence during 10 field expeditions carried out between 2015 and 2018 in southern Amazonia, the arc of deforestation, Brazil.

Here, we present 192 new records of 22 primate species in 56 localities across the arc of deforestation (Fig. 1). Based on these data, we extend the ranges of *Alouatta puruensis*, *Ateles chamek*, and *Saimiri collinsi*; identify potential hybridization zones between *A. puruensis* and *A. discolor*, and between *At. chamek* and *At. marginatus*; redefine the range of *Plecturocebus moloch*; and clarify the ranges of *P. baptista* and *P. hoffmannsi*. Primates are valuable as flagship species for conservation (Dietz et al., 1994; Estrada et al., 2017; Chapman et al., 2020) and, as such, investments in research and conservation of the primate species from the arc of deforestation are expected to have a positive cascade effect on the protection of Amazonian biodiversity, ecosystem services, and mitigation of climate change.

## 2 Methods

From 2015 to 2018, 10 field expeditions were carried out in the arc of deforestation, southern Amazonia, across the states of Amazonas, Mato Grosso, Pará, and Rondônia in Brazil. All large-river interfluves were surveyed for the occurrence of primates as well as the interfluves of second-order rivers in the basins of the Xingu, Tapajós, Aripuanã, Ji-Paraná, Teles Pires, Juruena, Madeira, and Amazonas rivers. The expeditions lasted 21 d on average. A recording of *Mico marcai* long calls was used for playback in order to enhance the detection of marmosets by Rodrigo Costa-Araújo during active search for primates, which was carried out by foot and by boat along rivers. For each primate observation or vocalization, the species was recorded and the geographical coordinates were taken with a Garmin GPSMap 65s. For vocalization records, the identifications are based on both the long calls, which are genus-specific, and on the locality, for species-level distinctions. For observational records, the species identifications are based on the phenotype of the specimens. The identifications follow the latest review of the taxonomy and distribution of neotropical primates (Mittermeier et al., 2013) and the IUCN Red List (IUCN, 2022). All the records were transformed into decimal degrees using the speciesLink geographical coordinate converter (http://splink.cria.org.br/conversor?criaLANG=en, last access: 15 January 2023) and plotted on a map using QGIS (2023). This research did not involve animal experimentation, and therefore no ethical consent was required.

**Figure 2:**
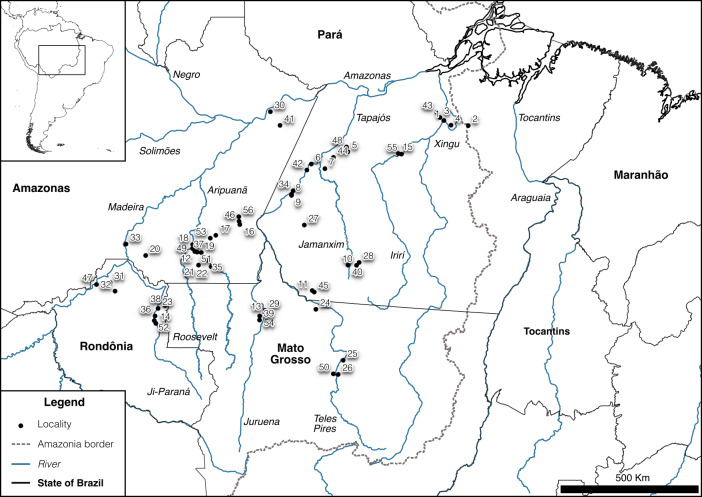
Map of the 56 localities where the 192 new occurrence records of primates were obtained during field expeditions between 2015 and 2018 in southern Amazonia, the arc of deforestation, Brazil (see Table 2). The localities group occurrence records under the threshold of a 10 km radius.

## 3 Results and discussion

We obtained 192 new occurrence records of 22 primate species and subspecies of *Alouatta*, *Aotus*, *Ateles*, *Cebus*, *Chiropotes*, *Lagothrix*, *Leontocebus*, *Pithecia*, *Plecturocebus*, *Saimiri*, and *Sapajus* (Tables 1 and 2; Supplement). Such records represent 56 localities (Fig. 2), which add to others obtained during the same field expeditions carried out in southern Amazonia between 2015 and 2018 (see Boubli et al., 2019; Costa-Araújo et al., 2019, 2021, 2022b, 2023; Byrne et al., 2021).

**Table 1:** Primate taxa recorded during 10 field expeditions carried out across the arc of deforestation, southern Amazonia, Brazil, between 2015 and 2018. The taxonomy follows Mittermeier et al. (2013) unless otherwise indicated, and the species conservation status follows the IUCN Red List (IUCN, 2022).

Species	Conservation status
*Alouatta belzebul* (Linnaeus, 1766)	Vulnerable
*Alouatta discolor* (Spix, 1823)	Vulnerable
*Alouatta puruensis*^1^ Lönnberg, 1941	Vulnerable
*Aotus azarae infulatus* (Kuhl, 1820)	Least Concern
*Ateles chamek* (Humboldt and Bonpland, 1811)	Endangered
*Ateles marginatus* (É. St.-Hilaire, 1809)	Endangered
*Cebus unicolor* Spix, 1823	Vulnerable
*Chiropotes albinasus* (I. St.-Hilaire and Deville, 1848)	Vulnerable
*Lagothrix cana cana* (É. St.-Hilaire in Humboldt and Bonpland, 1811)	Endangered
*Leontocebus weddelli weddelli*^2^ (Deville, 1849)	Least Concern
*Pithecia mittermeieri*^3^ Marsh, 2014	Vulnerable
*Plecturocebus baptista*^4^ (Lönnberg, 1939)	Least Concern
*Plecturocebus bernhardi*^4^ (M. Roosmalen, T. Roosmalen, and Mittermeier, 2002)	Least Concern
*Plecturocebus brunneus*^4^ (Wagner, 1842)	Vulnerable
*Plecturocebus cinerascens*^4^ (Spix, 1823)	Least Concern
*Plecturocebus grovesi*^4^ Boubli et al., 2019	Critically Endangered
*Plecturocebus hoffmannsi*^4^ (Thomas, 1908)	Least Concern
*Plecturocebus miltoni*^4^ (Dalponte, Silva, and Silva-Jr., 2014)	Data Deficient
*Plecturocebus moloch*^4^ (Hoffmannsegg, 1807)	Least Concern
*Saimiri collinsi* Osgood, 1916	Least Concern
*Saimiri ustus* I. St.-Hilaire, 1843	Near Threatened
*Sapajus apella apella* (Linnaeus, 1758)	Least Concern

^1^ Gregorin (2006); ^2^ Rylands et al. (2016); ^3^ Marsh (2014); ^4^ Byrne et al. (2016).

**Table 2:** List of 56 localities in decimal degrees, WGS84 ellipsoid, for the 192 new occurrence records of primates from the arc of deforestation, southern Amazonia, Brazil (see Fig. 2). The localities group occurrence records under the threshold of a 10 km radius.

Locality	Latitude	Longitude	Species
1	- 3.256066	- 52.101795	*Alouatta belzebul*
2	- 3.431207	- 51.273499	*A. belzebul*, *Sapajus apella apella*
3	- 3.154029	- 52.236515	*A. discolor*, *Ateles marginatus*, *S. a. apella*
4	- 3.415871	- 51.861140	*A. discolor*, *Plecturocebus moloch*, *Saimiri collinsi*, *S. a. apella*
5	- 4.321818	- 55.376763	*A. discolor*, *Sa. collinsi*, *S. a. apella*
6	- 4.739434	- 56.620976	*A. discolor*, *P. moloch*, *Sa. collinsi*, *S. a. apella*
7	- 4.896320	- 56.157203	*A. discolor*, *At. marginatus*, *Chiropotes albinasus*
8	- 5.766087	- 57.293432	*A. discolor*, *At. marginatus*, *S. a. apella*
9	- 5.806025	- 57.293842	*A. discolor*, *At. marginatus*, *C. albinasus*, *P. hoffmannsi*, *Sa. collinsi*, *S. a. apella*
10	- 8.188168	- 55.080342	*A. discolor*, *S. a. apella*
11	- 9.045870	- 56.589056	*A. discolor*, *At. marginatus*, *S. a. apella*
12	- 7.715710	- 60.587942	*A. puruensis*, *At. chamek*, *Sa. ustus*
13	- 9.810552	- 58.192113	*A. puruensis*, *C. albinasus*, *P. grovesi*, *Sa. ustus*, *S. a. apella*
14	- 10.162898	- 61.914299	*A. puruensis*, *Lagothrix cana cana*, *P. bernhardi*
15	- 4.381089	- 53.657286	*Aotus azarae infulatus*, *At. marginatus*, *C. albinasus*, *P. moloch*, *Sa. collinsi*, *S. a. apella*
16	- 6.798492	- 59.055495	*At. chamek*, *C. albinasus*, *P. cinerascens*
17	- 7.164853	- 59.873720	*At. chamek*, *C. albinasus*, *L. c. cana*
18	- 7.479167	- 60.660556	*At. chamek*, *P. cinerascens*
19	- 7.532118	- 60.474626	*At. chamek*, *Cebus unicolor*, *C. albinasus*, *L. c. cana*, *Pithecia mittermeieri*, *S. a. apella*
20	- 7.852230	- 62.262647	*At. chamek*, *L. c. cana*, *P. bernhardi*, *Sa. ustus*, *S. a. apella*
21	- 8.179956	- 60.461448	*At. chamek*, *P. miltoni*
22	- 8.232795	- 60.031846	*At. chamek*, *C. albinasus*, *Pi. mittermeieri*, *P. miltoni*, *Sa. ustus*, *S. a. apella*
23	- 9.655326	- 61.838057	*At. chamek*
24	- 9.683964	- 56.465441	*At. chamek*, *P. grovesi*, *S. a. apella*
25	- 11.420282	- 55.535212	*At. chamek*, *At. marginatus*, *Sapajus a. apella*
26	- 11.900939	- 55.704336	*At. chamek*, *S. a. apella*
27	- 6.816851	- 56.858321	*At. marginatus*, *S. a. apella*
28	- 8.089851	- 54.991485	*At. marginatus*, *C. albinasus*, *P. moloch*
29	- 9.718700	- 58.195878	*At. chamek*, *S. a. apella*
30	- 2.956981	- 58.014683	*L. c. cana*, *Sa. ustus*
31	- 8.769543	- 63.475592	*Leontocebus weddelli weddelli*, *P. brunneus*
32	- 9.066980	- 63.304194	*Le. weddelli weddelli*, *P. brunneus*, *Sa. ustus*, *S. a. apella*
33	- 7.463472	- 62.932391	*Pi. mittermeieri*, *P. bernhardi*
34	- 5.649224	- 57.239382	*At. marginatus*
35	- 8.018963	- 60.136411	*C. albinasus*
36	- 9.911144	- 61.942478	*C. albinasus*
37	- 7.753777	- 60.366578	*L. c. cana*
38	- 9.557020	- 61.604184	*L. c. cana*
39	- 10.050181	- 58.383100	*Pi. mittermeieri*
40	- 8.181511	- 55.369755	*At. marginatus*, *C. albinasus*, *Sa. collinsi; S. a. apella*
41	- 3.422861	- 57.685694	*P. baptista*
42	- 4.942477	- 56.770202	*P. hoffmannsi*
43	- 2.969967	- 52.349933	*P. moloch*
44	- 4.512367	- 55.866501	*Sa. collinsi*
45	- 9.096743	- 56.520596	*At. marginatus*, *C. albinasus*, *P. moloch*, *Sa. collinsi*
46	- 6.687303	- 59.080460	*Sa. ustus*
47	- 8.839395	- 63.937128	*Sa. ustus*
48	- 4.158472	- 55.420808	*S. a. apella*
49	- 7.614548	- 60.665216	*Ce. unicolor*, *C. albinasus; L. c. cana*
50	- 11.881577	- 55.868323	*S. a. apella*
51	- 7.736875	- 60.510270	*L. c. cana*, *Pi. mittermeieri*, *P. miltoni*
52	- 10.070073	- 61.969437	*L. c. cana*, *P. bernhardi*, *S. a. apella*
53	- 7.262815	- 60.061120	*Pi. mittermeieri*, *P. cinerascens*, *S. a. apella*
54	- 9.911395	- 58.377819	*P. cinerascens*, *Sa. ustus*, *S. a. apella*
55	- 4.400933	- 53.548912	*Sa. collinsi*, *S. a. apella*
56	- 6.531828	- 59.089128	*L. c. cana*, *Sa. ustus*

**Figure 3:**
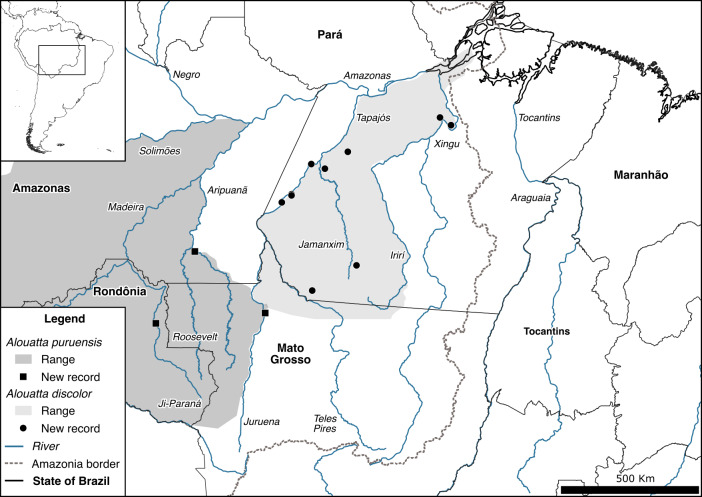
Geographic distributions and new records of *Alouatta puruensis* and *Alouatta discolor* in southern Amazonia, the arc of deforestation, Brazil, with extension of the geographic distribution of *A. puruensis* into the right margin of the Juruena River, within 20 km of the range of *A. discolor*.

**Figure 4:**
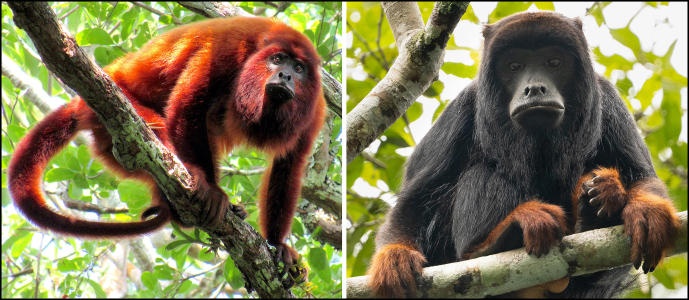
Left: adult female of *Alouatta puruensis* recorded on the right bank of the middle Juruena River, Mato Grosso, Brazil (credit: Rodrigo Costa-Araújo). Right: adult male of *Alouatta discolor* (credit: Jéssica dos Anjos).

Based on current knowledge of the taxonomy and distribution of neotropical primates (Mittermeier et al., 2013; IUCN, 2022), our records clarify the range and population dynamics of eight species: *Alouatta puruensis* Lönnberg, 1941, *A. discolor* (Spix, 1823), *Ateles chamek* (Humboldt and Bonpland, 1811), *At. marginatus* (St.-Hilaire, 1809), *Plecturocebus moloch* (Hoffmannsegg, 1807), *P. hoffmannsi* (Thomas, 1908), *P. baptista* (Lönnberg, 1939), and *Saimiri collinsi* Osgood, 1916. One of our records of *Alouatta puruensis* is on the right bank of the middle Juruena River, which extends the species range into the Juruena–Teles Pires interfluve, only 20 km from the southwestern limit of the distribution of *A. discolor* (Fig. 3). Although there is a record of sympatry between these two *Alouatta* species in the Juruena–Teles Pires interfluve (Paranaíta municipality; Pinto and Setz, 2000), the only species currently considered to occur in this region is *A. discolor* (Gregorin, 2006). Additionally, we suggest the existence of a natural hybridization zone across the middle Juruena River, with bidirectional dispersal of and introgression in these two *Alouatta* species, i.e., dispersal of *A. puruensis* individuals from the left margin and introgression into *A. discolor* populations in the right margin and vice versa, using the large islands of the middle Juruena River as stepping stones. Howler monkeys can swim across rivers (Gonzalez-Socoloske and Snarr, 2010) and are common on Amazonian fluvial islands (Rabelo et al., 2019). Moreover, introgressive hybridization is relatively common in primates (Cortés-Ortiz et al., 2019a) and is known for howler monkey species with contrasting phenotypes (Cortés-Ortiz et al., 2019b; Mourthé et al., 2019), like *A. puruensis* and *A. discolor* (Fig. 4).

**Figure 5:**
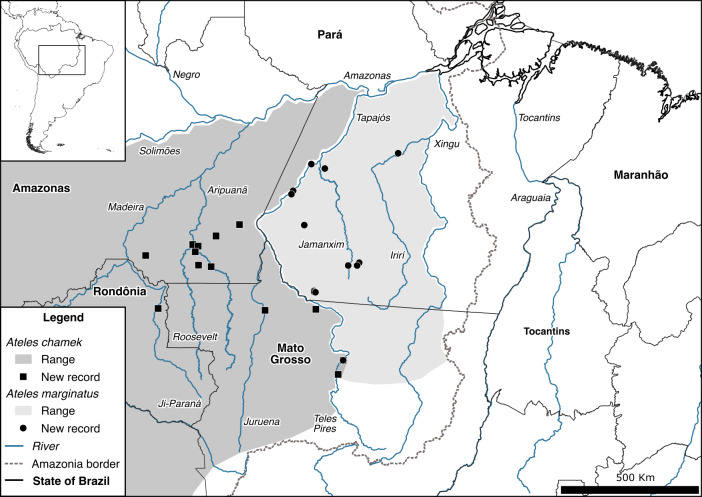
Geographic distributions and new records of *Ateles chamek* and *Ateles marginatus* in southern Amazonia, the arc of deforestation, Brazil, with extension of the geographic distribution of *At. chamek* into the right margin of the Teles Pires River, within the range of *At. marginatus*.

Two of our records of *Ateles chamek* are on the right margin of the Teles Pires River, within the range of *At. marginatus* (Fig. 5), as also reported by Lazari et al. (2020). In this region, we observed adult spider monkeys completely black in pelage, lacking the white hairs on the forehead and cheeks that are characteristic of *At. marginatus*. Therefore, we identified these individuals as *At. chamek*. Moreover, one of the two records of *At. chamek* overlaps with a record of *At. marginatus*. Similarly to the case of *A. puruensis* and *A. discolor* in the middle Juruena River, we suggest ongoing, bidirectional dispersal of and gene flow between *At. chamek* and *At. marginatus* across the middle Teles Pires River, with fluvial islands as stepping stones. Spider monkeys are also known to swim (Nunes, 2014; Chaves and Stoner, 2010) and can use human-made structures, such as raft cables, to cross rivers (Lazari et al., 2020).

**Figure 6:**
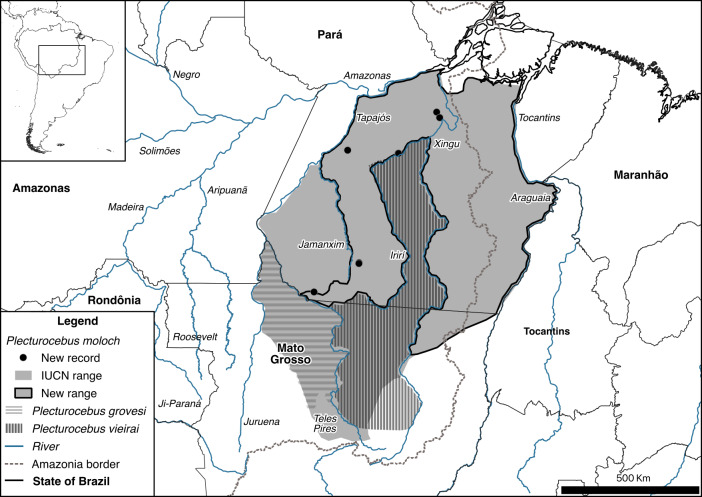
New geographic distribution and new records of *Plecturocebus moloch* together with distributions of *Plecturocebus grovesi* and *Plecturocebus vieirai* in southern Amazonia, the arc of deforestation, Brazil.

We propose a new delimitation for the distribution range of *P. moloch* (Fig. 6) based on our records and on the exclusion of the ranges of *P. grovesi* and *P. vieirai* (Gualda-Barrros, Nascimento and Amaral, 2012) (Costa-Araújo et al., 2022b). *Plecturocebus grovesi* and *P. vieirai* were recently described for titi monkeys within the range formerly thought to belong to *P. moloch* (Hershkovitz, 1990; Roosmalen et al., 2002). Nonetheless, it remains necessary to determine the type locality of *P. moloch* and the validity of two of its synonyms – *P. remulus* (Thomas, 1908) and *P. emiliae* (Thomas, 1911) (Lönnberg, 1939; Hershkovitz, 1990) – and to clarify the taxonomy of titis from the Jamanxim–Teles Pires interfluve.

**Figure 7:**
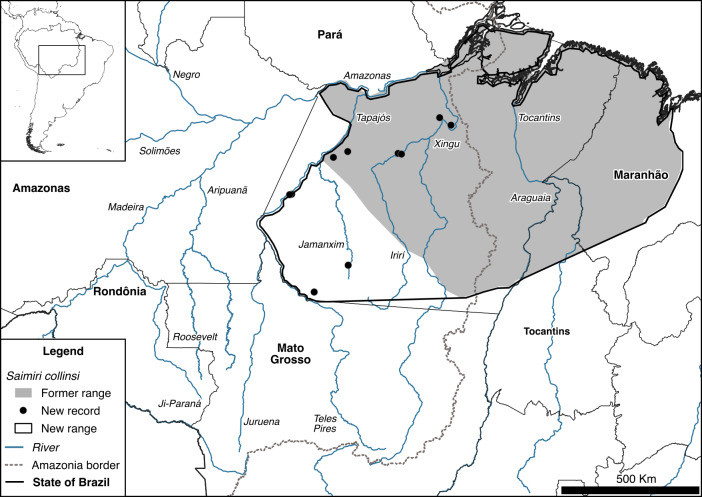
New hypothesis for the geographic distribution of *Saimiri collinsi* in southern Amazonia, arc of deforestation, Brazil.

We found a group of *P. baptista* within the range of *P. hoffmannsi*. Our observations reinforce previous findings of Rocha et al. (2019) and Printes et al. (2018). Although *Plecturocebus baptista* and *P. hoffmannsi* are easily distinguished by their pelage color, we suggest that their phenotypes might be a clinal variation of a single species as is currently proposed for *P. parecis* and *P. cinerascens* (Byrne et al., 2021). This suggestion follows the fact that the taxonomy and distribution of *P. baptista* and *P. hoffmannsi* are little studied, they have not been subjected to molecular phylogenetic analyses to date, and there are no physical barriers impeding gene flow between these species. Finally, we recorded *Saimiri collinsi* on the left bank of the Jamanxim River and on the right bank of the upper Tapajós and lower Teles Pires rivers, extending the species range by approximately 600 km to the west (Fig. 7).

## 4 Conclusion

Considering our findings and the new hypotheses raised here, new field expeditions are necessary for obtaining further occurrence records, specimens, and genetic samples for research on the taxonomy, distribution, population dynamics, and evolutionary history of the primate species from the arc of deforestation – especially in areas poorly or never explored to date. Such basic knowledge is also paramount for biodiversity protection because species are the main subjects of awareness-raising and conservation strategies, and the lack of information on their taxonomy and distribution impedes effective conservation measurements (Costa et al., 2005; Rylands and Mittermeier, 2014). The implementation and improvement of conservation strategies envisioned in the National Action Plan for the Conservation of Amazonian Primates (ICMBio, 2017), for example, depend on such basic information.

Additional records, specimens, and samples of *A. puruensis* and *A. discolor* in the middle Juruena River and of *At. chamek* and *At. marginatus* in the middle Teles Pires River would allow us to refine the taxonomy and the extent of occurrence of these species to confirm the existence of gene flow and to characterize the population dynamics in these hybrid zones. Such field data and materials are also necessary to clarify the taxonomy, distribution, and evolutionary history of *P. baptista*, *P. hoffmannsi*, and *P. moloch*. Given the scarcity of information on and the pace of habitat loss faced by the primates in the arc of deforestation, this should be a priority for research and conservation among platyrrhines.

## Supplement

10.5194/pb-11-1-2024-supplement The dataset with 192 new occurrence records of 22 primate species of Alouatta, Aotus, Ateles, Cebus, Chiropotes, Lagothrix, Leontocebus, Pithecia, Plecturocebus, Saimiri, and Sapajus genera can be accessed via the Supplement. The supplement related to this article is available online at: https://doi.org/10.5194/pb-11-1-2024-supplement.

## Data Availability

No datasets were used in this article.
