# Supplementary material for: A dataset of new occurrence records of primates from the arc of deforestation, Brazil
Source: Primate Biol. 2024 Jan 22;11(1):1–11. doi: 10.5194/pb-11-1-2024 (PMC11262026; doi:10.5194/pb-11-1-2024)
Supplement: The dataset with 192 new occurrence records of 22 primate species of Alouatta, Aotus, Ateles, Cebus, Chiropotes, Lagothrix, Leontocebus, Pithecia, Plecturocebus, Saimiri, and Sapajus genera can be accessed via the Supplement. The supplement related to this article is available online at: https://doi [file pb-11-1-supplement.pdf]

Supplement of Primate Biol., 11, 1–11, 2024
https://doi.org/10.5194/pb-11-1-2024-supplement
© Author(s) 2024. CC BY 4.0 License.

Primate Biology
Open Access

Supplement of

A dataset of new occurrence records of primates from the arc of deforestation, Brazil

Rodrigo Costa-Araújo et al.

Correspondence to: Rodrigo Costa-Araújo (rcostaaraujo@dpz.eu)

The copyright of individual parts of the supplement might differ from the article licence.

Supplementary Material. Dataset of 192 new occurrence records (decimal degrees, WGS 84 ellipsoid) of 22 primate species and subspecies of *Alouatta*, *Aotus*, *Ateles*, *Cebus*, *Chiropotes*, *Lagothrix*, *Leontocebus*, *Pithecia*, *Plecturocebus*, *Saimiri*, and *Sapajus* genera from the arc of deforestation, southern Amazonia, Brazil.

Species	Latitude	Longitude	State	Municipality	Type of record
Alouatta belzebul	-3,256066	-52,101795	Pará	Altamira	observation
Alouatta belzebul	-3,431207	-51,273499	Pará	Anapu	observation
Alouatta discolor	-3,154029	-52,236515	Pará	Altamira	vocalization
Alouatta discolor	-3,415871	-51,861140	Pará	Vitória do Xingu	vocalization
Alouatta discolor	-4,321818	-55,376763	Pará	Rurópolis	observation
Alouatta discolor	-4,739434	-56,620976	Pará	Itaituba	observation
Alouatta discolor	-4,896320	-56,157203	Pará	Trairão	observation
Alouatta discolor	-5,766087	-57,293432	Pará	Jacareacanga	observation
Alouatta discolor	-5,806025	-57,293842	Pará	Jacareacanga	observation
Alouatta discolor	-6,043952	-57,625627	Pará	Jacareacanga	vocalization
Alouatta discolor	-8,188168	-55,080342	Pará	Jacareacanga	observation
Alouatta discolor	-9,045870	-56,589056	Pará	Jacareacanga	observation
Alouatta puruensis	-7,715710	-60,587942	Amazonas	Novo Aripuanã	observation
Alouatta puruensis	-9,810552	-58,192113	Mato Grosso	Nova Bandeirantes	observation
Alouatta puruensis	-10,162898	-61,914299	Rondônia	Ouro Preto do Oeste	observation
Aotus azarae infulatus	-4,381089	-53,657286	Pará	Altamira	observation
Ateles chamek	-6,798492	-59,055495	Amazonas	Apuí	observation
Ateles chamek	-6,799800	-59,066411	Amazonas	Apuí	observation
Ateles chamek	-7,164853	-59,873720	Amazonas	Apuí	observation
Ateles chamek	-7,190935	-59,866180	Amazonas	Apuí	observation
Ateles chamek	-7,479167	-60,660556	Amazonas	Apuí	observation
Ateles chamek	-7,532118	-60,474626	Amazonas	Apuí	observation
Ateles chamek	-7,724356	-60,570772	Amazonas	Apuí	observation
Ateles chamek	-7,852230	-62,262647	Amazonas	Humaitá	observation
Ateles chamek	-8,179956	-60,461448	Amazonas	Novo Aripuanã	observation
Ateles chamek	-8,232795	-60,031846	Amazonas	Apuí	observation
Ateles chamek	-9,655326	-61,838057	Rondônia	Vale do Anari	observation

Species	Latitude	Longitude	State	Municipality	Type of record
Ateles chamek	-9,683964	-56,465441	Mato Grosso	Paranaíta	observation
Ateles chamek	-9,718700	-58,195878	Mato Grosso	Nova Bandeirantes	observation
Ateles chamek	-11,420282	-55,535212	Mato Grosso	Sinop	observation
Ateles chamek	-11,900939	-55,704336	Mato Grosso	Sorriso	observation
Ateles marginatus	-4,371760	-53,661465	Pará	Altamira	observation
Ateles marginatus	-4,740682	-56,619694	Pará	Itaituba	observation
Ateles marginatus	-4,891533	-56,158671	Pará	Trairão	observation
Ateles marginatus	-5,649224	-57,239382	Pará	Itaituba	observation
Ateles marginatus	-5,764504	-57,296657	Pará	Jacareacanga	observation
Ateles marginatus	-6,816851	-56,858321	Pará	Jacareacanga	observation
Ateles marginatus	-8,089851	-54,991485	Pará	Altamira	observation
Ateles marginatus	-8,097535	-54,989894	Pará	Altamira	observation
Ateles marginatus	-8,181511	-55,369755	Pará	Novo Progresso	observation
Ateles marginatus	-8,191731	-55,068541	Pará	Altamira	observation
Ateles marginatus	-8,193925	-55,059866	Pará	Altamira	observation
Ateles marginatus	-8,194418	-55,362012	Pará	Novo Progresso	observation
Ateles marginatus	-9,059008	-56,531378	Pará	Jacareacanga	observation
Ateles marginatus	-9,096743	-56,520596	Pará	Jacareacanga	observation
Ateles marginatus	-9,104173	-56,477179	Pará	Jacareacanga	observation
Ateles marginatus	-11,420268	-55,535448	Mato Grosso	Sinop	observation
Cebus unicolor	-7,494167	-60,482322	Amazonas	Apuí	observation
Cebus unicolor	-7,614548	-60,665216	Amazonas	Apuí	observation
Chiropotes albinasus	-4,371760	-53,661465	Pará	Altamira	vocalization
Chiropotes albinasus	-4,377047	-53,664946	Pará	Altamira	observation
Chiropotes albinasus	-4,740857	-56,619720	Pará	Itaituba	observation
Chiropotes albinasus	-4,740935	-56,638851	Pará	Itaituba	observation
Chiropotes albinasus	-5,769441	-57,291435	Pará	Jacareacanga	observation
Chiropotes albinasus	-5,781748	-57,261809	Pará	Itaituba	observation
Chiropotes albinasus	-6,785360	-59,049433	Amazonas	Apuí	observation
Chiropotes albinasus	-6,798525	-59,055482	Amazonas	Apuí	observation

Species	Latitude	Longitude	State	Municipality	Type of record
Chiropotes albinasus	-7,190322	-59,864595	Amazonas	Apuí	observation
Chiropotes albinasus	-7,484720	-60,477481	Amazonas	Apuí	observation
Chiropotes albinasus	-7,501831	-60,481858	Amazonas	Apuí	observation
Chiropotes albinasus	-7,576732	-60,671287	Amazonas	Apuí	observation
Chiropotes albinasus	-7,612338	-60,664355	Amazonas	Apuí	observation
Chiropotes albinasus	-8,018963	-60,136411	Amazonas	Apuí	observation
Chiropotes albinasus	-8,125647	-54,998307	Pará	Altamira	observation
Chiropotes albinasus	-8,183114	-55,365275	Pará	Novo Progresso	observation
Chiropotes albinasus	-8,231920	-60,029423	Amazonas	Apuí	observation
Chiropotes albinasus	-8,232070	-60,033680	Amazonas	Apuí	observation
Chiropotes albinasus	-9,096868	-56,520560	Pará	Jacareacanga	observation
Chiropotes albinasus	-9,845558	-58,235430	Mato Grosso	Cotriguaçu	observation
Chiropotes albinasus	-9,911144	-61,942478	Rondônia	Vale do Anari	observation
Lagothrix cana cana	-2,956981	-58,014683	Amazonas	Urucurituba	observation
Lagothrix cana cana	-6,531828	-59,089128	Amazonas	Apuí	observation
Lagothrix cana cana	-7,163810	-59,871508	Amazonas	Apuí	observation
Lagothrix cana cana	-7,194129	-59,864045	Amazonas	Apuí	observation
Lagothrix cana cana	-7,529642	-60,475932	Amazonas	Apuí	observation
Lagothrix cana cana	-7,612338	-60,664355	Amazonas	Apuí	observation
Lagothrix cana cana	-7,736875	-60,510270	Amazonas	Apuí	observation
Lagothrix cana cana	-7,753777	-60,366578	Amazonas	Apuí	observation
Lagothrix cana cana	-7,773209	-60,564321	Amazonas	Apuí	observation
Lagothrix cana cana	-7,845560	-62,306463	Amazonas	Humaitá	observation
Lagothrix cana cana	-9,557020	-61,604184	Rondônia	Vale do Anari	observation
Lagothrix cana cana	-10,070073	-61,969437	Rondônia	Ji-Paraná	observation
Lagothrix cana cana	-10,122558	-61,902792	Rondônia	Ji-Paraná	vocalization
Leontocebus weddelli weddelli	-8,769543	-63,475592	Rondônia	Candeias do Jamari	observation
Leontocebus weddelli weddelli	-9,066980	-63,304194	Rondônia	Itapuã do Oeste	observation
Pithecia mittermeieri	-7,262815	-60,061120	Amazonas	Apuí	observation
Pithecia mittermeieri	-7,463472	-62,932391	Amazonas	Humaitá	observation

Species	Latitude	Longitude	State	Municipality	Type of record
Pithecia mittermeieri	-7,530242	-60,475596	Amazonas	Apuí	observation
Pithecia mittermeieri	-7,736256	-60,515709	Amazonas	Apuí	observation
Pithecia mittermeieri	-8,232976	-60,030847	Amazonas	Apuí	observation
Pithecia mittermeieri	-10,050181	-58,383100	Mato Grosso	Cotriguaçu	observation
Plecturocebus baptista	-3,422861	-57,685694	Amazonas	Maués	observation
Plecturocebus bernhardi	-7,456201	-62,939055	Amazonas	Humaitá	observation
Plecturocebus bernhardi	-7,462714	-62,932357	Amazonas	Humaitá	observation
Plecturocebus bernhardi	-7,838514	-62,293781	Amazonas	Humaitá	observation
Plecturocebus bernhardi	-7,859012	-62,258619	Amazonas	Humaitá	observation
Plecturocebus bernhardi	-10,063351	-61,960871	Rondônia	Ji-Paraná	observation
Plecturocebus bernhardi	-10,124698	-61,908830	Rondônia	Ji-Paraná	observation
Plecturocebus bernhardi	-10,144905	-61,907793	Rondônia	Ji-Paraná	observation
Plecturocebus brunneus	-8,769542	-63,475592	Rondônia	Candeias do Jamari	observation
Plecturocebus brunneus	-8,769543	-63,475592	Rondônia	Candeias do Jamari	observation
Plecturocebus brunneus	-9,066980	-63,304194	Rondônia	Itapuã do Oeste	observation
Plecturocebus cinerascens	-6,799215	-59,055320	Amazonas	Apuí	observation
Plecturocebus cinerascens	-7,204571	-60,018139	Amazonas	Apuí	observation
Plecturocebus cinerascens	-7,519522	-60,653148	Amazonas	Apuí	observation
Plecturocebus cinerascens	-7,526585	-60,653049	Amazonas	Apuí	vocalization
Plecturocebus cinerascens	-9,911395	-58,377819	Mato Grosso	Cotriguaçu	observation
Plecturocebus grovesi	-9,667538	-56,496601	Mato Grosso	Paranaíta	observation
Plecturocebus grovesi	-9,791949	-58,195175	Mato Grosso	Nova Bandeirantes	vocalization
Plecturocebus hoffmannsi	-5,741886	-57,361631	Pará	Jacareacanga	observation
Plecturocebus hoffmannsi	-4,942477	-56,770202	Pará	Itaituba	observation
Plecturocebus miltoni	-7,795544	-60,537823	Amazonas	Apuí	observation
Plecturocebus miltoni	-8,172043	-60,453659	Amazonas	Novo Aripuanã	observation
Plecturocebus miltoni	-8,176226	-60,450408	Amazonas	Apuí	observation
Plecturocebus miltoni	-8,178109	-60,455417	Amazonas	Novo Aripuanã	observation
Plecturocebus miltoni	-8,220575	-60,025239	Amazonas	Apuí	observation
Plecturocebus miltoni	-8,226501	-60,029947	Amazonas	Apuí	observation

Species	Latitude	Longitude	State	Municipality	Type of record
Plecturocebus moloch	-2,969967	-52,349933	Pará	Altamira	observation
Plecturocebus moloch	-3,157132	-52,242953	Pará	Altamira	observation
Plecturocebus moloch	-3,159748	-52,248455	Pará	Altamira	observation
Plecturocebus moloch	-4,268417	-55,382044	Pará	Rurópolis	observation
Plecturocebus moloch	-4,268418	-55,382045	Pará	Rurópolis	observation
Plecturocebus moloch	-4,368058	-53,649794	Pará	Altamira	observation
Plecturocebus moloch	-4,370280	-53,658815	Pará	Altamira	observation
Plecturocebus moloch	-4,372368	-53,662517	Pará	Altamira	observation
Plecturocebus moloch	-4,376896	-53,649244	Pará	Altamira	observation
Plecturocebus moloch	-8,098461	-54,994222	Pará	Altamira	observation
Plecturocebus moloch	-9,094306	-56,527142	Pará	Jacareacanga	observation
Plecturocebus moloch	-8,098461	-54,994223	Pará	Altamira	observation
Plecturocebus moloch	-8,125651	-54,998562	Pará	Altamira	observation
Saimiri collinsi	-3,159748	-52,248455	Pará	Altamira	observation
Saimiri collinsi	-3,414968	-51,862088	Pará	Vitória do Xingu	observation
Saimiri collinsi	-4,313622	-55,379458	Pará	Rurópolis	observation
Saimiri collinsi	-4,377047	-53,664946	Pará	Altamira	observation
Saimiri collinsi	-4,400933	-53,548912	Pará	Altamira	observation
Saimiri collinsi	-4,512367	-55,866501	Pará	Itaituba	observation
Saimiri collinsi	-5,767912	-57,290098	Pará	Jacareacanga	observation
Saimiri collinsi	-5,781224	-57,358087	Pará	Jacareacanga	observation
Saimiri collinsi	-8,183433	-55,363710	Pará	Novo Progresso	observation
Saimiri collinsi	-9,095654	-56,522555	Pará	Jacareacanga	observation
Saimiri ustus	-2,952100	-58,068181	Amazonas	Urucurituba	observation
Saimiri ustus	-6,573147	-59,081904	Amazonas	Apuí	observation
Saimiri ustus	-6,687303	-59,080460	Amazonas	Apuí	observation
Saimiri ustus	-7,722717	-60,574936	Amazonas	Apuí	observation
Saimiri ustus	-7,856605	-62,262775	Amazonas	Humaitá	observation
Saimiri ustus	-8,227582	-60,032353	Amazonas	Apuí	observation
Saimiri ustus	-8,839395	-63,937128	Rondônia	Porto Velho	observation

Species	Latitude	Longitude	State	Municipality	Type of record
Saimiri ustus	-9,067489	-63,304714	Rondônia	Itapuã do Oeste	observation
Saimiri ustus	-9,844137	-58,233728	Mato Grosso	Cotriguaçu	observation
Saimiri ustus	-9,848728	-58,269594	Mato Grosso	Cotriguaçu	observation
Saimiri ustus	-9,863258	-58,257559	Mato Grosso	Cotriguaçu	observation
Saimiri ustus	-9,868031	-58,328950	Mato Grosso	Cotriguaçu	observation
Sapajus apella apella	-3,153210	-52,238517	Pará	Altamira	observation
Sapajus apella apella	-3,414968	-51,862088	Pará	Vitória do Xingu	observation
Sapajus apella apella	-3,433550	-51,272531	Pará	Anapu	observation
Sapajus apella apella	-4,158472	-55,420808	Pará	Rurópolis	observation
Sapajus apella apella	-4,311525	-55,379610	Pará	Rurópolis	observation
Sapajus apella apella	-4,313622	-55,379458	Pará	Rurópolis	observation
Sapajus apella apella	-4,400933	-53,548912	Pará	Altamira	observation
Sapajus apella apella	-4,435548	-53,628088	Pará	Altamira	observation
Sapajus apella apella	-4,438924	-53,621913	Pará	Altamira	observation
Sapajus apella apella	-4,439817	-53,623675	Pará	Altamira	observation
Sapajus apella apella	-4,884648	-56,187969	Pará	Trairão	observation
Sapajus apella apella	-4,890649	-56,156454	Pará	Trairão	observation
Sapajus apella apella	-5,781852	-57,268322	Pará	Itaituba	observation
Sapajus apella apella	-5,783523	-57,256974	Pará	Itaituba	observation
Sapajus apella apella	-5,805452	-57,292603	Pará	Jacareacanga	observation
Sapajus apella apella	-6,121724	-57,589114	Pará	Jacareacanga	observation
Sapajus apella apella	-6,812682	-56,855636	Pará	Jacareacanga	observation
Sapajus apella apella	-7,268159	-60,057074	Amazonas	Apuí	observation
Sapajus apella apella	-7,470663	-60,494342	Amazonas	Apuí	observation
Sapajus apella apella	-7,836196	-62,303610	Amazonas	Humaitá	observation
Sapajus apella apella	-8,195322	-55,366323	Pará	Novo Progresso	observation
Sapajus apella apella	-8,211416	-60,020714	Amazonas	Apuí	observation
Sapajus apella apella	-8,215703	-55,014563	Pará	Altamira	observation
Sapajus apella apella	-8,227582	-60,032353	Amazonas	Apuí	observation
Sapajus apella apella	-8,232947	-60,029154	Amazonas	Apuí	observation

Species	Latitude	Longitude	State	Municipality	Type of record
Sapajus apella apella	-9,063070	-56,592167	Pará	Jacareacanga	vocalization
Sapajus apella apella	-9,067489	-63,304714	Rondônia	Itapuã do Oeste	observation
Sapajus apella apella	-9,682842	-56,465968	Mato Grosso	Paranaíta	observation
Sapajus apella apella	-9,727820	-58,175349	Mato Grosso	Nova Bandeirantes	observation
Sapajus apella apella	-9,730757	-58,210872	Mato Grosso	Nova Bandeirantes	observation
Sapajus apella apella	-9,846406	-58,236330	Mato Grosso	Cotriguaçu	observation
Sapajus apella apella	-9,848728	-58,269594	Mato Grosso	Cotriguaçu	observation
Sapajus apella apella	-9,856182	-58,260230	Mato Grosso	Cotriguaçu	observation
Sapajus apella apella	-9,863445	-58,328272	Mato Grosso	Cotriguaçu	observation
Sapajus apella apella	-9,898467	-58,323943	Mato Grosso	Cotriguaçu	observation
Sapajus apella apella	-10,080955	-61,937099	Rondônia	Ji-Paraná	observation
Sapajus apella apella	-11,420268	-55,535448	Mato Grosso	Sinop	observation
Sapajus apella apella	-11,420282	-55,535212	Mato Grosso	Sinop	observation
Sapajus apella apella	-11,881577	-55,868323	Mato Grosso	Sorriso	observation
Sapajus apella apella	-11,900939	-55,704336	Mato Grosso	Sorriso	observation
